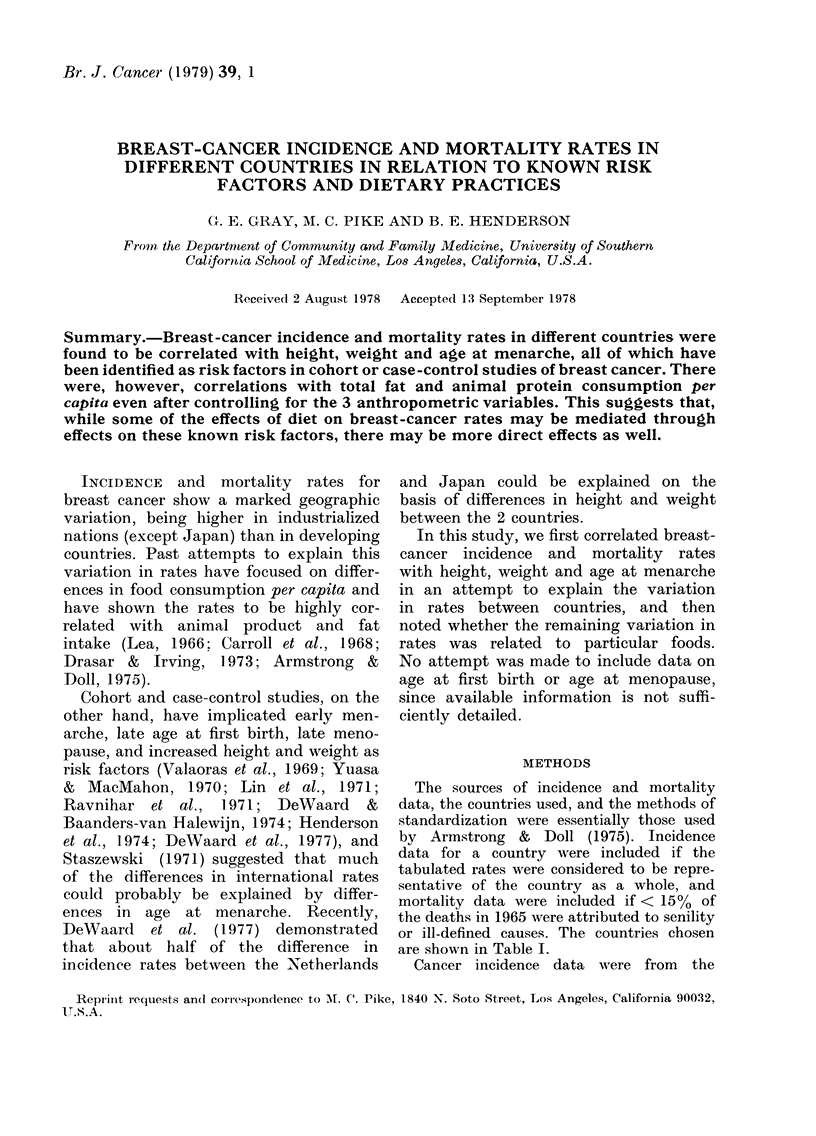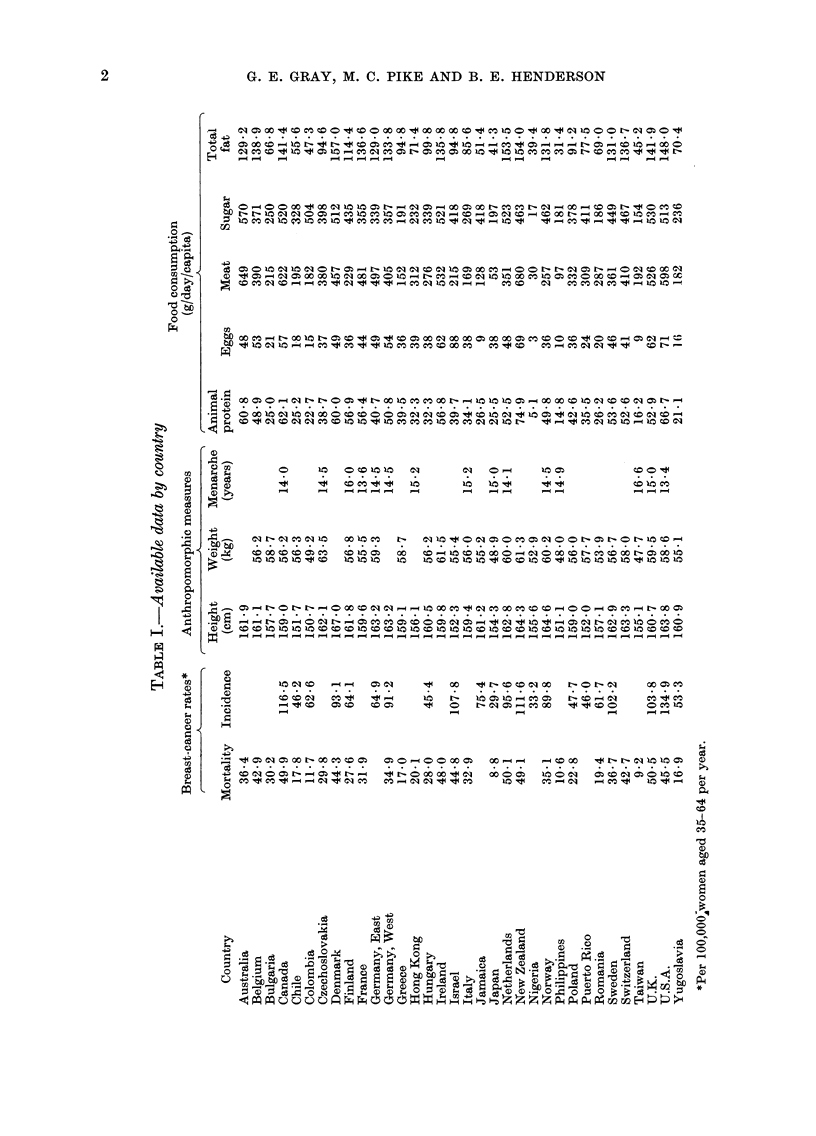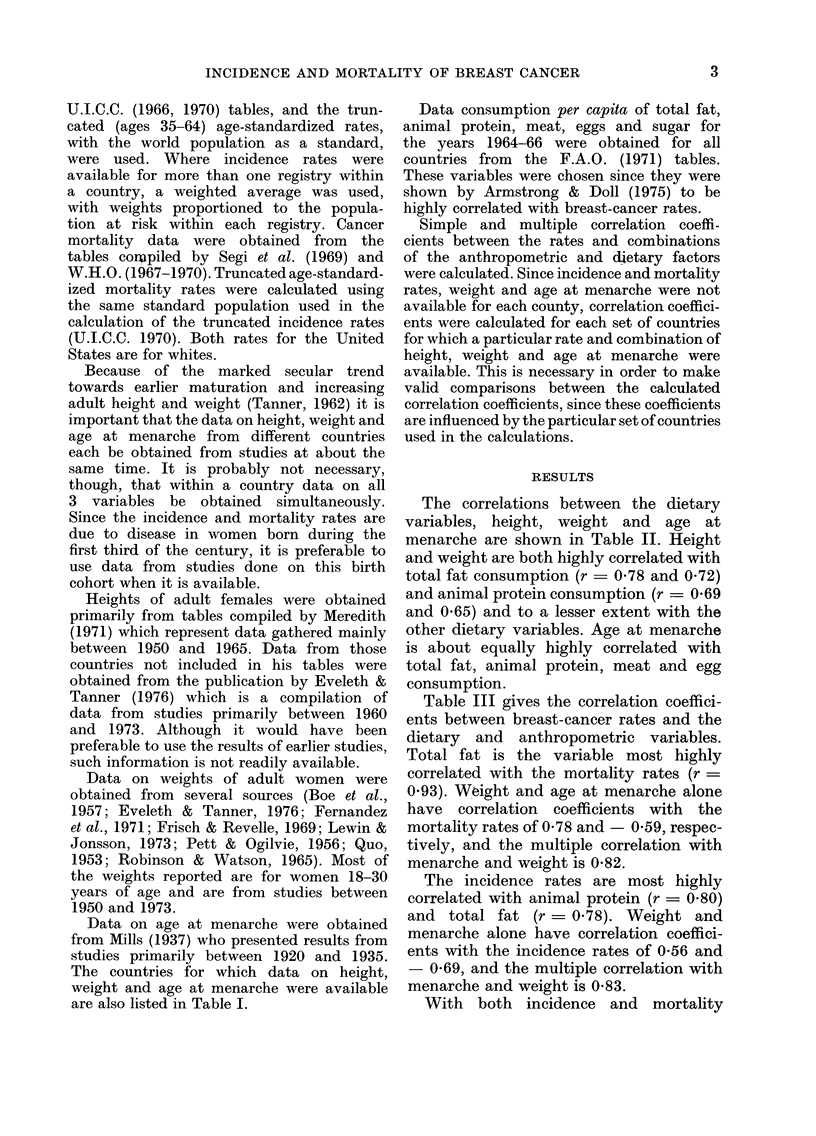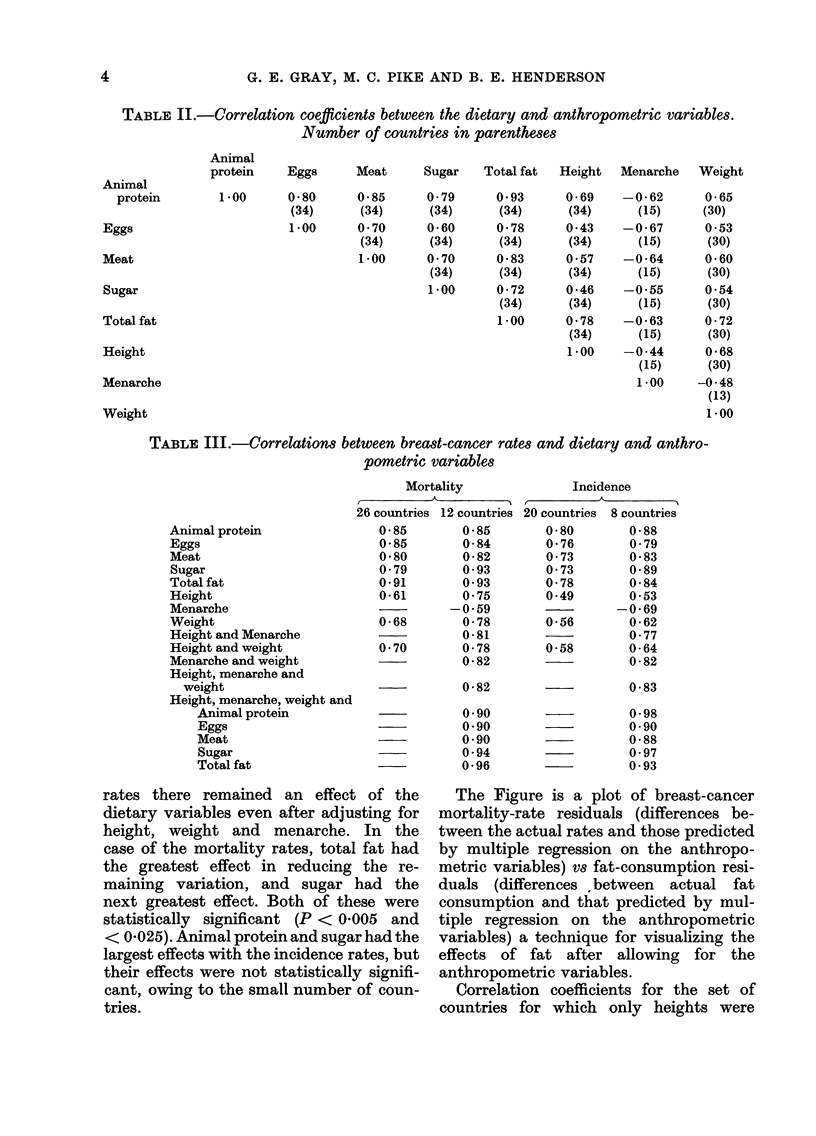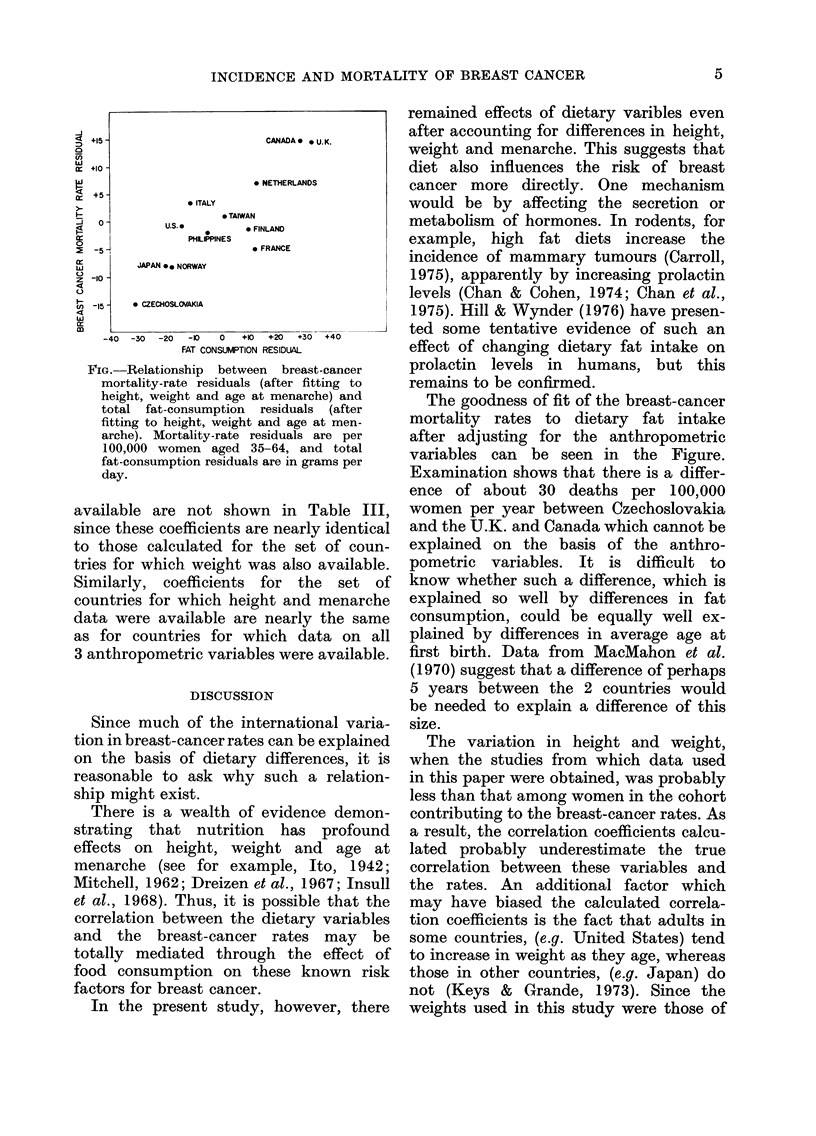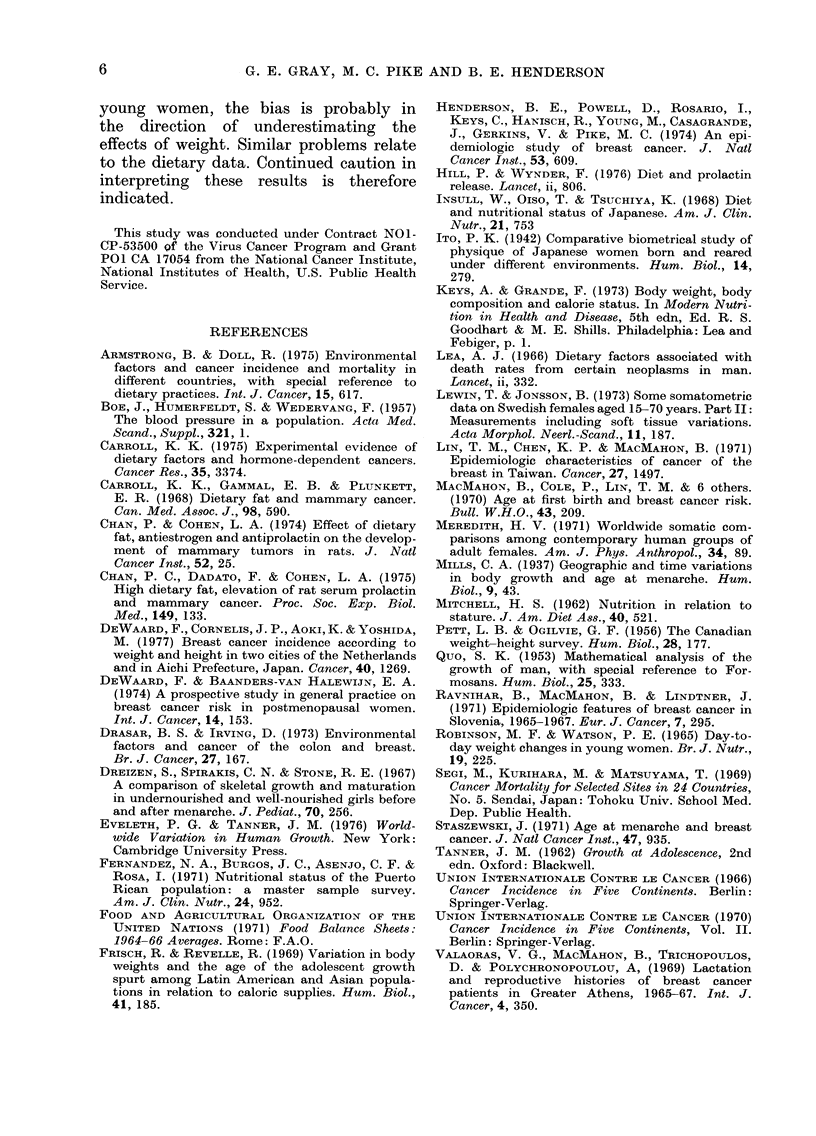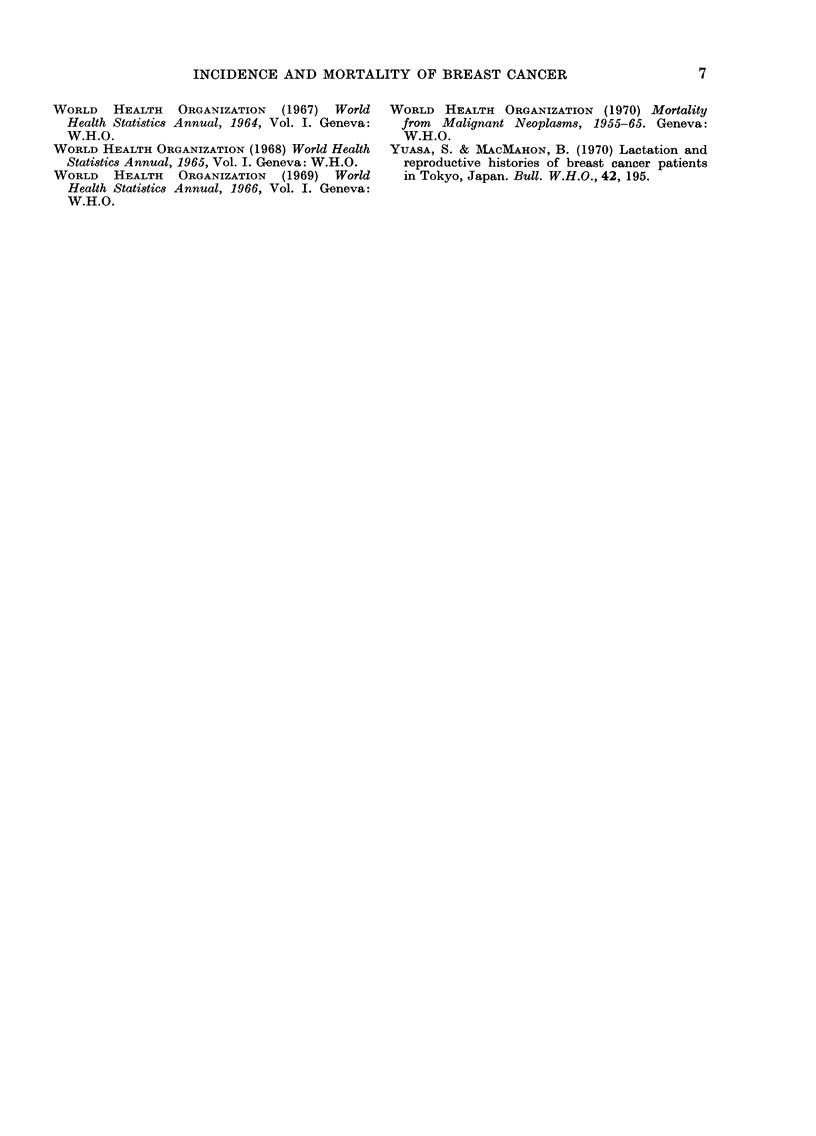# Breast-cancer incidence and mortality rates in different countries in relation to known risk factors and dietary practices.

**DOI:** 10.1038/bjc.1979.1

**Published:** 1979-01

**Authors:** G. E. Gray, M. C. Pike, B. E. Henderson

## Abstract

Breast-cancer incidence and mortality rates in different countries were found to be correlated with height, weight and age at menarche, all of which have been identified as risk factors in cohort or case-control studies of breast cancer. There were, however, correlations with total fat and animal protein consumption per capita even after controlling for the 3 anthropometric variables. This suggests that, while some of the effects of diet on breast-cancer rates may be mediated through effects on these known risk factors, there may be more direct effects as well.


*Br. J. Cancer* (1979) **39**, 1

### BREAST-CANCER INCIDENCE AND MORTALITY RATES IN DIFFERENT COUNTRIES IN RELATION TO KNOWN RISK FACTORS AND DIETARY PRACTICES

G. E. GRAY, M. C. PIKE AND B. E. HENDERSON

*From the Department of Community and Family Medicine, University of Southern California School of Medicine, Los Angeles, California, U.S.A.*

Received 2 August 1978 Accepted 13 September 1978

**Summary.—Breast-cancer incidence and mortality rates in different countries were found to be correlated with height, weight and age at menarche, all of which have been identified as risk factors in cohort or case-control studies of breast cancer. There were, however, correlations with total fat and animal protein consumption *per capita* even after controlling for the 3 anthropometric variables. This suggests that, while some of the effects of diet on breast-cancer rates may be mediated through effects on these known risk factors, there may be more direct effects as well.**

INCIDENCE and mortality rates for breast cancer show a marked geographic variation, being higher in industrialized nations (except Japan) than in developing countries. Past attempts to explain this variation in rates have focused on differences in food consumption *per capita* and have shown the rates to be highly correlated with animal product and fat intake (Lea, 1966; Carroll *et al.*, 1968; Drasar & Irving, 1973; Armstrong & Doll, 1975).

Cohort and case-control studies, on the other hand, have implicated early menarche, late age at first birth, late menopause, and increased height and weight as risk factors (Valaoras *et al.*, 1969; Yuasa & MacMahon, 1970; Lin *et al.*, 1971; Ravnihar *et al.*, 1971; DeWaard & Baanders-van Halewijn, 1974; Henderson *et al.*, 1974; DeWaard *et al.*, 1977), and Staszewski (1971) suggested that much of the differences in international rates could probably be explained by differences in age at menarche. Recently, DeWaard *et al.* (1977) demonstrated that about half of the difference in incidence rates between the Netherlands and Japan could be explained on the basis of differences in height and weight between the 2 countries.

In this study, we first correlated breast-cancer incidence and mortality rates with height, weight and age at menarche in an attempt to explain the variation in rates between countries, and then noted whether the remaining variation in rates was related to particular foods. No attempt was made to include data on age at first birth or age at menopause, since available information is not sufficiently detailed.

### METHODS

The sources of incidence and mortality data, the countries used, and the methods of standardization were essentially those used by Armstrong & Doll (1975). Incidence data for a country were included if the tabulated rates were considered to be representative of the country as a whole, and mortality data were included if $< 15\%$ of the deaths in 1965 were attributed to senility or ill-defined causes. The countries chosen are shown in Table I.

Cancer incidence data were from the

Reprint requests and correspondence to M. C. Pike, 1840 N. Soto Street, Los Angeles, California 90032, U.S.A.

TABLE I.—*Available data by country*

| | Breast-cancer rates* | | Anthropomorphic measures | | | Food consumption (g/day/capita) | | | | |
|---|---|---|---|---|---|---|---|---|---|---|
| Country | Mortality | Incidence | Height (cm) | Weight (kg) | Menarche (years) | Animal protein | Eggs | Meat | Sugar | Total fat |
| Australia | 36·4 | | 161·9 | | | 60·8 | 48 | 649 | 570 | 129·2 |
| Belgium | 42·9 | | 161·1 | 56·2 | | 48·9 | 53 | 390 | 371 | 138·9 |
| Bulgaria | 30·2 | | 157·7 | 58·7 | | 25·0 | 21 | 215 | 250 | 66·8 |
| Canada | 49·9 | 116·5 | 159·0 | 56·2 | 14·0 | 62·1 | 57 | 622 | 520 | 141·4 |
| Chile | 17·8 | 46·2 | 151·7 | 56·3 | | 25·2 | 18 | 195 | 328 | 55·6 |
| Colombia | 11·7 | 62·6 | 150·7 | 49·2 | | 22·7 | 15 | 182 | 504 | 47·3 |
| Czechoslovakia | 29·8 | | 162·1 | 63·5 | 14·5 | 38·7 | 37 | 380 | 398 | 94·6 |
| Denmark | 44·3 | 93·1 | 167·0 | | | 60·0 | 49 | 457 | 512 | 157·0 |
| Finland | 27·6 | 64·1 | 161·8 | 56·8 | 16·0 | 56·9 | 36 | 229 | 435 | 114·4 |
| France | 31·9 | | 159·6 | 55·5 | 13·6 | 56·4 | 44 | 481 | 355 | 136·6 |
| Germany, East | | 64·9 | 163·2 | 59·3 | 14·5 | 40·7 | 49 | 497 | 339 | 129·0 |
| Germany, West | 34·9 | 91·2 | 163·2 | | 14·5 | 50·8 | 54 | 405 | 357 | 133·8 |
| Greece | 17·0 | | 159·1 | 58·7 | | 39·5 | 36 | 152 | 191 | 94·8 |
| Hong Kong | 20·1 | | 156·1 | | 15·2 | 32·3 | 39 | 312 | 232 | 71·4 |
| Hungary | 28·0 | 45·4 | 160·5 | 56·2 | | 32·3 | 38 | 276 | 339 | 99·8 |
| Ireland | 48·0 | | 159·8 | 61·5 | | 56·8 | 62 | 532 | 521 | 135·8 |
| Israel | 44·8 | 107·8 | 152·3 | 55·4 | | 39·7 | 88 | 215 | 418 | 94·8 |
| Italy | 32·9 | | 159·4 | 56·0 | 15·2 | 34·1 | 38 | 169 | 269 | 85·6 |
| Jamaica | | 75·4 | 161·2 | 55·2 | | 26·5 | 9 | 128 | 418 | 51·4 |
| Japan | 8·8 | 29·7 | 154·3 | 48·9 | 15·0 | 25·5 | 38 | 53 | 197 | 41·3 |
| Netherlands | 50·1 | 95·6 | 162·8 | 60·0 | 14·1 | 52·5 | 48 | 351 | 523 | 153·5 |
| New Zealand | 49·1 | 111·6 | 164·3 | 61·3 | | 74·9 | 69 | 680 | 463 | 154·0 |
| Nigeria | | 33·2 | 155·6 | 52·9 | | 5·1 | 3 | 30 | 17 | 39·4 |
| Norway | 35·1 | 89·8 | 164·6 | 60·2 | 14·5 | 49·8 | 36 | 257 | 462 | 131·8 |
| Philippines | 10·6 | | 151·1 | 48·0 | 14·9 | 14·8 | 10 | 97 | 181 | 31·4 |
| Poland | 22·8 | 47·7 | 159·0 | 56·0 | | 42·6 | 36 | 332 | 378 | 91·2 |
| Puerto Rico | | 46·0 | 152·0 | 57·7 | | 35·5 | 24 | 309 | 411 | 77·5 |
| Romania | 19·4 | 61·7 | 157·1 | 53·9 | | 26·2 | 20 | 287 | 186 | 69·0 |
| Sweden | 36·7 | 102·2 | 162·9 | 56·7 | | 53·6 | 46 | 361 | 449 | 131·0 |
| Switzerland | 42·7 | | 163·3 | 58·0 | | 52·6 | 41 | 410 | 467 | 136·7 |
| Taiwan | 9·2 | | 155·1 | 47·7 | 16·6 | 16·2 | 9 | 192 | 154 | 45·2 |
| U.K. | 50·5 | 103·8 | 160·7 | 59·5 | 15·0 | 52·9 | 62 | 526 | 530 | 141·9 |
| U.S.A. | 45·5 | 134·9 | 163·8 | 58·6 | 13·4 | 66·7 | 71 | 598 | 513 | 148·0 |
| Yugoslavia | 16·9 | 53·3 | 160·9 | 55·1 | | 21·1 | 16 | 182 | 236 | 70·4 |

*Per 100,000 women aged 35–64 per year.

U.I.C.C. (1966, 1970) tables, and the truncated (ages 35–64) age-standardized rates, with the world population as a standard, were used. Where incidence rates were available for more than one registry within a country, a weighted average was used, with weights proportioned to the population at risk within each registry. Cancer mortality data were obtained from the tables compiled by Segi *et al.* (1969) and W.H.O. (1967–1970). Truncated age-standardized mortality rates were calculated using the same standard population used in the calculation of the truncated incidence rates (U.I.C.C. 1970). Both rates for the United States are for whites.

Because of the marked secular trend towards earlier maturation and increasing adult height and weight (Tanner, 1962) it is important that the data on height, weight and age at menarche from different countries each be obtained from studies at about the same time. It is probably not necessary, though, that within a country data on all 3 variables be obtained simultaneously. Since the incidence and mortality rates are due to disease in women born during the first third of the century, it is preferable to use data from studies done on this birth cohort when it is available.

Heights of adult females were obtained primarily from tables compiled by Meredith (1971) which represent data gathered mainly between 1950 and 1965. Data from those countries not included in his tables were obtained from the publication by Eveleth & Tanner (1976) which is a compilation of data from studies primarily between 1960 and 1973. Although it would have been preferable to use the results of earlier studies, such information is not readily available.

Data on weights of adult women were obtained from several sources (Boe *et al.*, 1957; Eveleth & Tanner, 1976; Fernandez *et al.*, 1971; Frisch & Revelle, 1969; Lewin & Jonsson, 1973; Pett & Ogilvie, 1956; Quo, 1953; Robinson & Watson, 1965). Most of the weights reported are for women 18–30 years of age and are from studies between 1950 and 1973.

Data on age at menarche were obtained from Mills (1937) who presented results from studies primarily between 1920 and 1935. The countries for which data on height, weight and age at menarche were available are also listed in Table I.

Data consumption *per capita* of total fat, animal protein, meat, eggs and sugar for the years 1964–66 were obtained for all countries from the F.A.O. (1971) tables. These variables were chosen since they were shown by Armstrong & Doll (1975) to be highly correlated with breast-cancer rates.

Simple and multiple correlation coefficients between the rates and combinations of the anthropometric and dietary factors were calculated. Since incidence and mortality rates, weight and age at menarche were not available for each county, correlation coefficients were calculated for each set of countries for which a particular rate and combination of height, weight and age at menarche were available. This is necessary in order to make valid comparisons between the calculated correlation coefficients, since these coefficients are influenced by the particular set of countries used in the calculations.

### RESULTS

The correlations between the dietary variables, height, weight and age at menarche are shown in Table II. Height and weight are both highly correlated with total fat consumption ($r = 0{\cdot}78$ and 0·72) and animal protein consumption ($r = 0{\cdot}69$ and 0·65) and to a lesser extent with the other dietary variables. Age at menarche is about equally highly correlated with total fat, animal protein, meat and egg consumption.

Table III gives the correlation coefficients between breast-cancer rates and the dietary and anthropometric variables. Total fat is the variable most highly correlated with the mortality rates ($r = 0{\cdot}93$). Weight and age at menarche alone have correlation coefficients with the mortality rates of 0·78 and − 0·59, respectively, and the multiple correlation with menarche and weight is 0·82.

The incidence rates are most highly correlated with animal protein ($r = 0{\cdot}80$) and total fat ($r = 0{\cdot}78$). Weight and menarche alone have correlation coefficients with the incidence rates of 0·56 and − 0·69, and the multiple correlation with menarche and weight is 0·83.

With both incidence and mortality

TABLE II.—*Correlation coefficients between the dietary and anthropometric variables. Number of countries in parentheses*

| | Animal protein | Eggs | Meat | Sugar | Total fat | Height | Menarche | Weight |
|---|---|---|---|---|---|---|---|---|
| Animal protein | 1·00 | 0·80 (34) | 0·85 (34) | 0·79 (34) | 0·93 (34) | 0·69 (34) | −0·62 (15) | 0·65 (30) |
| Eggs | | 1·00 | 0·70 (34) | 0·60 (34) | 0·78 (34) | 0·43 (34) | −0·67 (15) | 0·53 (30) |
| Meat | | | 1·00 | 0·70 (34) | 0·83 (34) | 0·57 (34) | −0·64 (15) | 0·60 (30) |
| Sugar | | | | 1·00 | 0·72 (34) | 0·46 (34) | −0·55 (15) | 0·54 (30) |
| Total fat | | | | | 1·00 | 0·78 (34) | −0·63 (15) | 0·72 (30) |
| Height | | | | | | 1·00 | −0·44 (15) | 0·68 (30) |
| Menarche | | | | | | | 1·00 | −0·48 (13) |
| Weight | | | | | | | | 1·00 |

TABLE III.—*Correlations between breast-cancer rates and dietary and anthropometric variables*

| | Mortality | | Incidence | |
|---|---|---|---|---|
| | 26 countries | 12 countries | 20 countries | 8 countries |
| Animal protein | 0·85 | 0·85 | 0·80 | 0·88 |
| Eggs | 0·85 | 0·84 | 0·76 | 0·79 |
| Meat | 0·80 | 0·82 | 0·73 | 0·83 |
| Sugar | 0·79 | 0·93 | 0·73 | 0·89 |
| Total fat | 0·91 | 0·93 | 0·78 | 0·84 |
| Height | 0·61 | 0·75 | 0·49 | 0·53 |
| Menarche | —— | −0·59 | —— | −0·69 |
| Weight | 0·68 | 0·78 | 0·56 | 0·62 |
| Height and Menarche | —— | 0·81 | —— | 0·77 |
| Height and weight | 0·70 | 0·78 | 0·58 | 0·64 |
| Menarche and weight | —— | 0·82 | —— | 0·82 |
| Height, menarche and weight | —— | 0·82 | —— | 0·83 |
| Height, menarche, weight and | | | | |
| Animal protein | —— | 0·90 | —— | 0·98 |
| Eggs | —— | 0·90 | —— | 0·90 |
| Meat | —— | 0·90 | —— | 0·88 |
| Sugar | —— | 0·94 | —— | 0·97 |
| Total fat | —— | 0·96 | —— | 0·93 |

rates there remained an effect of the dietary variables even after adjusting for height, weight and menarche. In the case of the mortality rates, total fat had the greatest effect in reducing the remaining variation, and sugar had the next greatest effect. Both of these were statistically significant ($P < 0{\cdot}005$ and $< 0{\cdot}025$). Animal protein and sugar had the largest effects with the incidence rates, but their effects were not statistically significant, owing to the small number of countries.

The Figure is a plot of breast-cancer mortality-rate residuals (differences between the actual rates and those predicted by multiple regression on the anthropometric variables) *vs* fat-consumption residuals (differences between actual fat consumption and that predicted by multiple regression on the anthropometric variables) a technique for visualizing the effects of fat after allowing for the anthropometric variables.

Correlation coefficients for the set of countries for which only heights were

FIG.—Relationship between breast-cancer mortality-rate residuals (after fitting to height, weight and age at menarche) and total fat-consumption residuals (after fitting to height, weight and age at menarche). Mortality-rate residuals are per 100,000 women aged 35–64, and total fat-consumption residuals are in grams per day.

available are not shown in Table III, since these coefficients are nearly identical to those calculated for the set of countries for which weight was also available. Similarly, coefficients for the set of countries for which height and menarche data were available are nearly the same as for countries for which data on all 3 anthropometric variables were available.

### DISCUSSION

Since much of the international variation in breast-cancer rates can be explained on the basis of dietary differences, it is reasonable to ask why such a relationship might exist.

There is a wealth of evidence demonstrating that nutrition has profound effects on height, weight and age at menarche (see for example, Ito, 1942; Mitchell, 1962; Dreizen *et al.*, 1967; Insull *et al.*, 1968). Thus, it is possible that the correlation between the dietary variables and the breast-cancer rates may be totally mediated through the effect of food consumption on these known risk factors for breast cancer.

In the present study, however, there remained effects of dietary varibles even after accounting for differences in height, weight and menarche. This suggests that diet also influences the risk of breast cancer more directly. One mechanism would be by affecting the secretion or metabolism of hormones. In rodents, for example, high fat diets increase the incidence of mammary tumours (Carroll, 1975), apparently by increasing prolactin levels (Chan & Cohen, 1974; Chan *et al.*, 1975). Hill & Wynder (1976) have presented some tentative evidence of such an effect of changing dietary fat intake on prolactin levels in humans, but this remains to be confirmed.

The goodness of fit of the breast-cancer mortality rates to dietary fat intake after adjusting for the anthropometric variables can be seen in the Figure. Examination shows that there is a difference of about 30 deaths per 100,000 women per year between Czechoslovakia and the U.K. and Canada which cannot be explained on the basis of the anthropometric variables. It is difficult to know whether such a difference, which is explained so well by differences in fat consumption, could be equally well explained by differences in average age at first birth. Data from MacMahon *et al.* (1970) suggest that a difference of perhaps 5 years between the 2 countries would be needed to explain a difference of this size.

The variation in height and weight, when the studies from which data used in this paper were obtained, was probably less than that among women in the cohort contributing to the breast-cancer rates. As a result, the correlation coefficients calculated probably underestimate the true correlation between these variables and the rates. An additional factor which may have biased the calculated correlation coefficients is the fact that adults in some countries, (*e.g.* United States) tend to increase in weight as they age, whereas those in other countries, (*e.g.* Japan) do not (Keys & Grande, 1973). Since the weights used in this study were those of young women, the bias is probably in the direction of underestimating the effects of weight. Similar problems relate to the dietary data. Continued caution in interpreting these results is therefore indicated.

This study was conducted under Contract NO1-CP-53500 of the Virus Cancer Program and Grant PO1 CA 17054 from the National Cancer Institute, National Institutes of Health, U.S. Public Health Service.

### REFERENCES

ARMSTRONG, B. & DOLL, R. (1975) Environmental factors and cancer incidence and mortality in different countries, with special reference to dietary practices. *Int. J. Cancer*, **15**, 617.

BOE, J., HUMERFELDT, S. & WEDERVANG, F. (1957) The blood pressure in a population. *Acta Med. Scand., Suppl.*, **321**, 1.

CARROLL, K. K. (1975) Experimental evidence of dietary factors and hormone-dependent cancers. *Cancer Res.*, **35**, 3374.

CARROLL, K. K., GAMMAL, E. B. & PLUNKETT, E. R. (1968) Dietary fat and mammary cancer. *Can. Med. Assoc. J.*, **98**, 590.

CHAN, P. & COHEN, L. A. (1974) Effect of dietary fat, antiestrogen and antiprolactin on the development of mammary tumors in rats. *J. Natl Cancer Inst.*, **52**, 25.

CHAN, P. C., DADATO, F. & COHEN, L. A. (1975) High dietary fat, elevation of rat serum prolactin and mammary cancer. *Proc. Soc. Exp. Biol. Med.*, **149**, 133.

DEWAARD, F., CORNELIS, J. P., AOKI, K. & YOSHIDA, M. (1977) Breast cancer incidence according to weight and height in two cities of the Netherlands and in Aichi Prefecture, Japan. *Cancer*, **40**, 1269.

DEWAARD, F. & BAANDERS-VAN HALEWIJN, E. A. (1974) A prospective study in general practice on breast cancer risk in postmenopausal women. *Int. J. Cancer*, **14**, 153.

DRASAR, B. S. & IRVING, D. (1973) Environmental factors and cancer of the colon and breast. *Br. J. Cancer*, **27**, 167.

DREIZEN, S., SPIRAKIS, C. N. & STONE, R. E. (1967) A comparison of skeletal growth and maturation in undernourished and well-nourished girls before and after menarche. *J. Pediat.*, **70**, 256.

EVELETH, P. G. & TANNER, J. M. (1976) *World-wide Variation in Human Growth.* New York: Cambridge University Press.

FERNANDEZ, N. A., BURGOS, J. C., ASENJO, C. F. & ROSA, I. (1971) Nutritional status of the Puerto Rican population: a master sample survey. *Am. J. Clin. Nutr.*, **24**, 952.

FOOD AND AGRICULTURAL ORGANIZATION OF THE UNITED NATIONS (1971) *Food Balance Sheets: 1964–66 Averages.* Rome: F.A.O.

FRISCH, R. & REVELLE, R. (1969) Variation in body weights and the age of the adolescent growth spurt among Latin American and Asian populations in relation to caloric supplies. *Hum. Biol.*, **41**, 185.

HENDERSON, B. E., POWELL, D., ROSARIO, I., KEYS, C., HANISCH, R., YOUNG, M., CASAGRANDE, J., GERKINS, V. & PIKE, M. C. (1974) An epidemiologic study of breast cancer. *J. Natl Cancer Inst.*, **53**, 609.

HILL, P. & WYNDER, F. (1976) Diet and prolactin release. *Lancet*, ii, 806.

INSULL, W., OISO, T. & TSUCHIYA, K. (1968) Diet and nutritional status of Japanese. *Am. J. Clin. Nutr.*, **21**, 753

ITO, P. K. (1942) Comparative biometrical study of physique of Japanese women born and reared under different environments. *Hum. Biol.*, **14**, 279.

KEYS, A. & GRANDE, F. (1973) Body weight, body composition and calorie status. In *Modern Nutrition in Health and Disease*, 5th edn, Ed. R. S. Goodhart & M. E. Shills. Philadelphia: Lea and Febiger, p. 1.

LEA, A. J. (1966) Dietary factors associated with death rates from certain neoplasms in man. *Lancet*, ii, 332.

LEWIN, T. & JONSSON, B. (1973) Some somatometric data on Swedish females aged 15–70 years. Part II: Measurements including soft tissue variations. *Acta Morphol. Neerl.-Scand.*, **11**, 187.

LIN, T. M., CHEN, K. P. & MACMAHON, B. (1971) Epidemiologic characteristics of cancer of the breast in Taiwan. *Cancer*, **27**, 1497.

MACMAHON, B., COLE, P., LIN, T. M. & 6 others. (1970) Age at first birth and breast cancer risk. *Bull. W.H.O.*, **43**, 209.

MEREDITH, H. V. (1971) Worldwide somatic comparisons among contemporary human groups of adult females. *Am. J. Phys. Anthropol.*, **34**, 89.

MILLS, C. A. (1937) Geographic and time variations in body growth and age at menarche. *Hum. Biol.*, **9**, 43.

MITCHELL, H. S. (1962) Nutrition in relation to stature. *J. Am. Diet Ass.*, **40**, 521.

PETT, L. B. & OGILVIE, G. F. (1956) The Canadian weight–height survey. *Hum. Biol.*, **28**, 177.

QUO, S. K. (1953) Mathematical analysis of the growth of man, with special reference to Formosans. *Hum. Biol.*, **25**, 333.

RAVNIHAR, B., MACMAHON, B. & LINDTNER, J. (1971) Epidemiologic features of breast cancer in Slovenia, 1965–1967. *Eur. J. Cancer*, **7**, 295.

ROBINSON, M. F. & WATSON, P. E. (1965) Day-to-day weight changes in young women. *Br. J. Nutr.*, **19**, 225.

SEGI, M., KURIHARA, M. & MATSUYAMA, T. (1969) *Cancer Mortality for Selected Sites in 24 Countries*, No. 5. Sendai, Japan: Tohoku Univ. School Med. Dep. Public Health.

STASZEWSKI, J. (1971) Age at menarche and breast cancer. *J. Natl Cancer Inst.*, **47**, 935.

TANNER, J. M. (1962) *Growth at Adolescence*, 2nd edn. Oxford: Blackwell.

UNION INTERNATIONALE CONTRE LE CANCER (1966) *Cancer Incidence in Five Continents.* Berlin: Springer-Verlag.

UNION INTERNATIONALE CONTRE LE CANCER (1970) *Cancer Incidence in Five Continents*, Vol. II. Berlin: Springer-Verlag.

VALAORAS, V. G., MACMAHON, B., TRICHOPOULOS, D. & POLYCHRONOPOULOU, A, (1969) Lactation and reproductive histories of breast cancer patients in Greater Athens, 1965–67. *Int. J. Cancer*, **4**, 350.

WORLD HEALTH ORGANIZATION (1967) *World Health Statistics Annual, 1964*, Vol. I. Geneva: W.H.O.

WORLD HEALTH ORGANIZATION (1968) *World Health Statistics Annual, 1965*, Vol. I. Geneva: W.H.O.

WORLD HEALTH ORGANIZATION (1969) *World Health Statistics Annual, 1966*, Vol. I. Geneva: W.H.O.

WORLD HEALTH ORGANIZATION (1970) *Mortality from Malignant Neoplasms, 1955–65*. Geneva: W.H.O.

YUASA, S. & MACMAHON, B. (1970) Lactation and reproductive histories of breast cancer patients in Tokyo, Japan. *Bull. W.H.O.*, **42**, 195.